# Corpora Cavernos invasion vs. Corpus Spongiosum invasion in Penile Cancer: A systematic review and meta-analysis

**DOI:** 10.7150/jca.56504

**Published:** 2021-01-30

**Authors:** Zaishang Li, Xueying Li, Wayne Lam, Yabing Cao, Jiunhung Geng, Antonio Augusto Ornellas, Fangjian Zhou, Hui Han

**Affiliations:** 1 Department of Urology, Shenzhen People's Hospital, The Second Clinic Medical College of Jinan University 518060, Shenzhen, Guangdong, P. R. China.; 2 Department of Urology, First Affiliated Hospital of Southern University of Science and Technology, 518060, Shenzhen, Guangdong, P. R. China.; 3 Department of Urology, Minimally Invasive Urology of Shenzhen research and Development Center of Medical Engineering and Technology, 518060, Shenzhen, Guangdong, P. R. China.; 4 Department of Oncology, The Seventh Affiliated Hospital Sun Yat-sen University, 518107, Shenzhen, Guangdong, P. R. China.; 5 Division of Urology, Department of Surgery, Queen Mary's Hospital, The University of Hong Kong, 999077, Hong Kong SAR, P. R. China.; 6 Department of Oncology, Hospital Kiang Wu, 999078, Macao SAR, P. R. China.; 7 Department of Urology, Kaohsiung Municipal Hsiao-Kang Hospital, 000800, Kaohsiung, Taiwan.; 8 Department of Urology, Kaohsiung Medical University Hospital, 000800, Kaohsiung, Taiwan.; 9 Kaohsiung Medical University, Kaohsiung, T 000800, Taiwan.; 10 Department of Urology, Brazilian National Institute of Cancer, 20230‑130, Rio de Janeiro, Brazil.; 11 Department of Urology, Sun Yat-sen University Cancer Center, 510060, Guangzhou, Guangdong, P. R. China.; 12 State Key Laboratory of Oncology in South China, 510060, Guangzhou, Guangdong, P. R. China.; 13 Collaborative Innovation Center of Cancer Medicine, 510060, Guangzhou, Guangdong, P. R. China.

**Keywords:** penile cancer, prognosis, meta-analysis, lymph node metastasis, stage

## Abstract

**Objective:** Changes were made in the 8^th^ edition of the American Joint Committee on Cancer (AJCC) staging system according to cavernosum invasion for penile squamous cell carcinoma. This study aimed to determine the difference of prognostic validity between corpora cavernosa (CC) invasion and corpus spongiosum (CS) invasion.

**Methods:** In this study, we searched PubMed, Cochrane CENTRAL, and Embase to select English-language articles until July 15, 2020. Pooled analyses of hazard ratios (HRs) and odds ratios (ORs) were performed.

**Results:** Eleven studies including 3692 cases were included in the final ananlysis (1431 cases with CC and 1360 cases with CS). According to the anatomical structure, the pooled results demonstrated that patients with CC invasion had a similar rate of LNM to those with CS invasion (OR 1.34, 95% confidence interval (CI) 0.97-1.86; *P*=0.076). However, patients with CC invasion had a higher rate of lymph node metastasis (LNM) than those with CS invasion according to the 8^th^ edition tumor stage (OR 1.58, 95% CI 1.14-2.21; *P*<0.001). Regarding survival, patients with CS invasion obtained a significantly better cancer-specific survival (CSS) (HR, 0.67; 95% CI, 0.46-0.96; *P*=0.030), but not in overall survival (OS) (HR: 1.30; 95% CI, 0.52-3.20; *P*=0.585) than those with CC invasion. No a significant publication bias was observed by Begg's and Egger's tests.

**Conclusions:** The systematic comparison suggests that patients with CS invasion had better CSS than those with CC invasion. CC invasion was associated with a high risk of LNM. The conclusions should be validated by large-scale studies.

## Introduction

The tumor stage of penile carcinoma is defined according to anatomy which has few changes 1-5. In 2008, Lejet et al. defined tumor involvement of the spongious and/or cavernous bodies as T2 or T3 which provides good prognostic stratification with a significant difference in survival 6. This classification has been proven to be a good way to distinguish the survival of penile patients in some studies and was accepted by the American Joint Committee on Cancer (AJCC) tumor-node-metastasis (TNM) staging system until 2018 5.

According to the latest TNM staging of penile carcinoma, the invasion of the corpus cavernosa (CC) and corpus spongiosum (CS) were classified as T3 and T2. Because of the incidence rate of penile cancer, there are few clinical data to verify the superiority of the 8th edition staging system 7-9. However, some studies confirmed that there is room for improvement in the 8th edition of T staging to predict the prognosis of patients more accurately 7-9.

A meta-analysis in 2019 showed that CC invasion and CS invasion were also significant predictors of inguinal lymph node metastasis (LNM) 10. However, this study failed to distinguish different anatomies and repeated statistics in the methods, so the results need further research.

To verify the prognostic value of the latest T stage, we performed a meta-analysis to explore the survival and LNM of CC invasion and CS invasion.

## Materials and methods

### Literature-search strategy

We searched the primary sources (PubMed, Embase, and the Cochrane Library) before July 2020. The following MeSH term was searched in [Title/Abstract]: penile cancer, penile tumor, penile neoplasm, penile squamous cell carcinoma, corpora cavernosa, corpora spongiosa, metastases, staging (eTable 1 in the Supplement).

### Inclusion and exclusion criteria

The inclusion criteria used were as follows: 1) determined the precise pathologically confirmed stage; 2) discriminated CC invasion and CS invasion; 3) reported the results of prognosis or LNM; and 4) written in English. Larger publications were included in the case of overlapping patient data from the same institution. If CC invasion and CS invasion were taken as a whole, the study was excluded.

### Data extraction and quality of data assessment

The reviewers (Zaishang Li and Xueying Li) independently extracted and summarized the information using the PICOS (Population, Intervention, Comparator, Outcome, and Study design) principle. A consensus meeting including a senior author (Hui Han) will be held to resolve any disagreements. The following information was extracted: year of publication, first author, country, recruitment period, sample size, age, follow-up time and outcome of the study. The quality assessment was performed according to the Cochrane Collaboration handbook 11. Any disagreements regarding the studies were resolved via discussion among all authors.

The quality of studies was assessed by the Newcastle-Ottawa Quality Assessment Scale. A score of 0-9 (allocated as stars), except randomized controlled trials.

### Statistical analysis

The survival rate of the patients affected was expressed as the hazard ratio (HR), and the presence of LNM was expressed as the odds ratio (OR). Engauge software was used to calculate the necessary data when survival data were not directly reported. For the rare cancer, T4 (number: Hölters S:2 and Wang: 2) was grouped together with T3, and these studies were also included in the final analysis 9, 12. One study was excluded from the survival analysis due to the limited number of patients (only 41 patients) 13. If I2 <40%, the fixed effects model was used; otherwise, the random-effects model was used. Stata version 12 (Stata Corp, College Station, TX, USA) and R 2.14.1 (http://www.r-proje ct.org) were used. Sensitivity analyses were performed for high-quality studies. Funnel plots were used to screen for potential publication bias. The significance level was set at 5% or a *P*-value<0.05.

## Results

### Summary of analyzed studies

**Figure 1:**
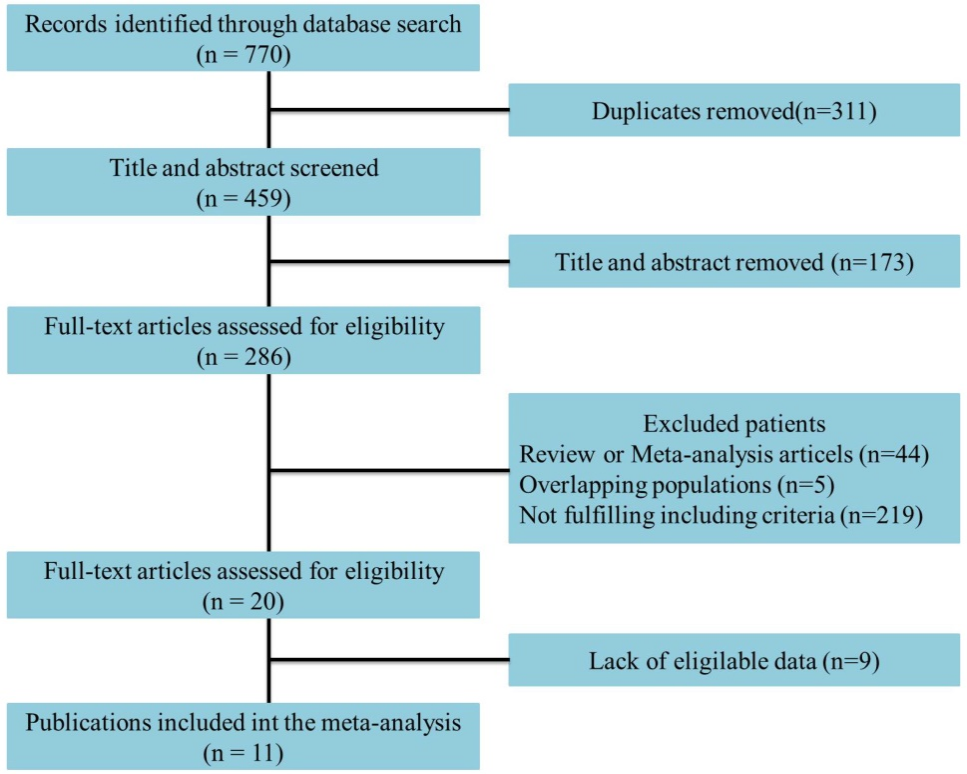
Flowchart of study selection.

**Figure 2:**
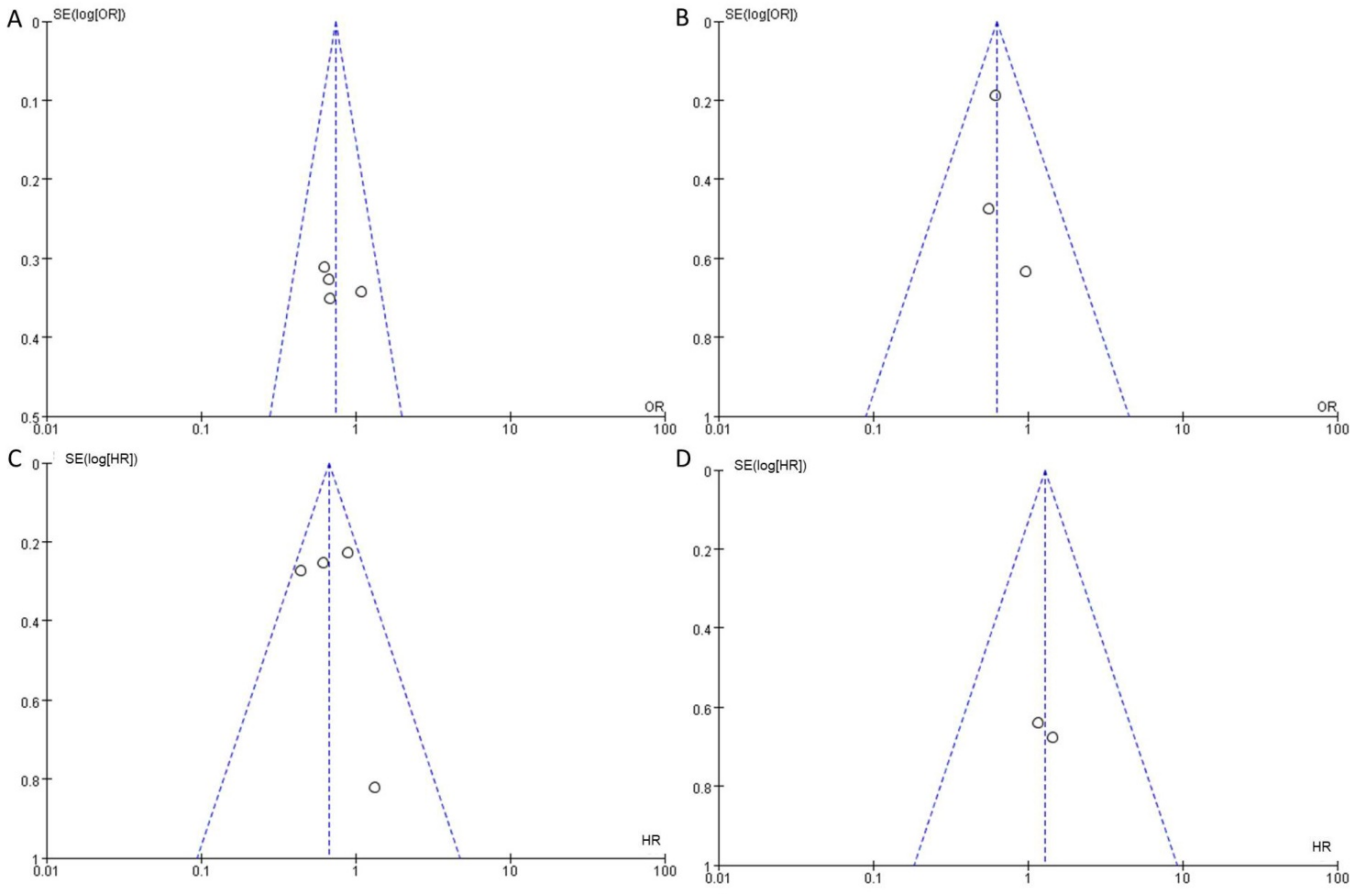
Funnel plots to detect the publication bias of eligible studies. A: according to the anatomical structure. B: according to the 8^th^ edition tumor stage. C: cancer-specific survival. D: overall survival.

Eleven studies incorporating 3692 cases met the final analysis criteria. The selection process is shown in Figure 1. Among these studies, LNM was investigated in 7 studies 8, 13-18, and survival was investigated in 4 studies 6, 7, 9, 12 (Table 1). Four studies classified LNM on the basis of the anatomical structure (CC invasion or CS invasion) 14-17, and 3 studies classified LNM on the basis of tumor stage (T2: tumor invading into CS or T3: tumor invading into CC) 8, 12, 13. The evaluation of publication bias was performed by Funnel plots, Begg's test and Egger's test (Figure 2,* eTable 1 in the Supplement*). The results of the sensitivity analysis are shown in *eTable 2 in the Supplement.*

### Association between LNM and cavernosum invasion in penile cancer patients

There were significant differences in anatomical structure and tumor stage. According to the anatomical structure, there was a partial intersection between CS invasion and CC invasion. However, there was no intersection between T2 and T3 according to the 8^th^ edition tumor stage. The associations between LNM and cavernosum invasion are presented separately in Table 2.

**Figure 3:**
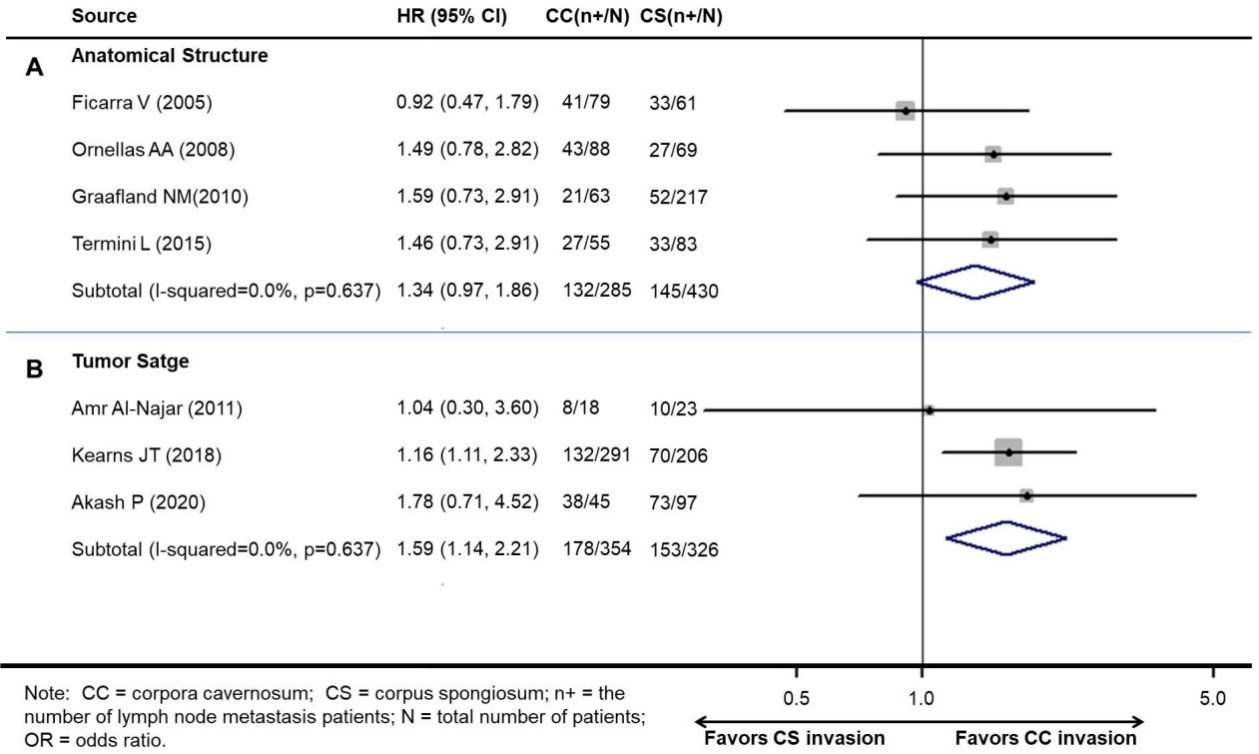
Meta-analysis of pooled odds ratios of association between LNM and cavernosum invasion in penile cancer. A: according to the anatomical structure. B: according to the 8^th^ edition tumor stage.

The pooled results demonstrated that patients with CS invasion had a similar rate of LNM to those with CC invasion according to the anatomical structure (OR 1.34, 95% CI 0.97-1.86;* P*=0.076, Figure 3A). The results show a growing trend towards LNM in patients with CS invasion according to the anatomical structure without statistical significance.

However, patients with CC invasion had a higher rate of LNM than those with CS invasion according to the 8^th^ edition tumor stage (OR 1.58, 95% CI 1.14-2.21; *P*<0.001) (Figure 3B).

### Association between survival and cavernosum invasion in penile cancer patient

Studies on survival analysis were allocated according to tumor stage. There was one study that contained two independent cohorts. Therefore, four cohorts reported the relationship between cavernosum invasion and cancer-specific survival (CSS), and two studies reported the relationship between cavernosum invasion and overall survival (OS).

**Figure 4:**
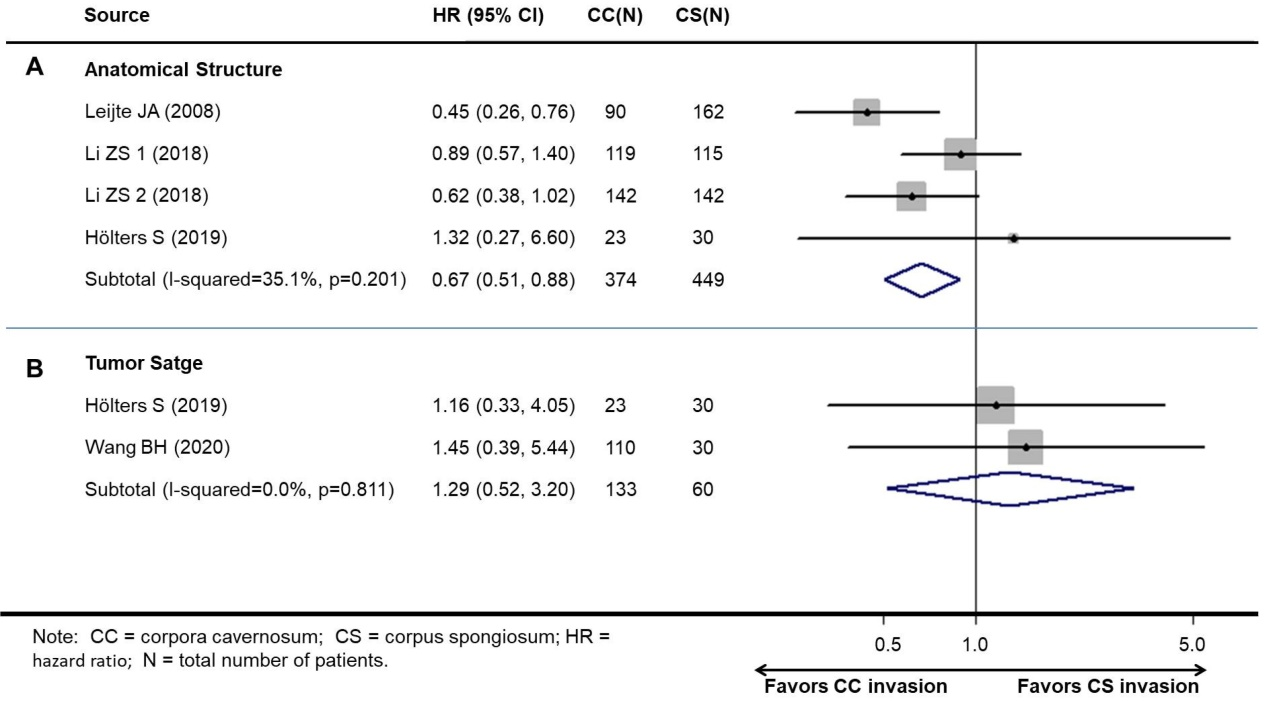
Meta-analysis of pooled hazard ratios of survival outcomes of cavernosum invasion in penile cancer patient. A: cancer-specific survival. B: overall survival.

Overall, patients with CS invasion obtained a significantly better CSS (HR, 0.67; 95% CI, 0.46-0.96; *P*=0.030, Figure 4A), but not OS (HR: 1.30; 95% CI, 0.52-3.20; *P*=0.585) than CC invasion (Figure 4B).

Preplanned subgroup analysis revealed the results of CC invasion compared with CC invasion (Table 3).

## Discussion

This study is the first systematic review to assess the difference between CS invasion and CC invasion in penile cancer. This study confirmed that patients with CS invasion have significantly higher CSS rates. However, no significant difference was found for OS between them. Interestingly, patients with CC invasion had a higher rate of LNM than those with CS invasion according to the 8^th^ edition tumor stage (OR 1.58, 95% CI 1.14-2.21; *P*<0.001). Simultaneously, there is a growing trend towards LNM in patients with CC invasion according to the anatomical structure (OR 1.34, 95% CI 0.97-1.86; *P*=0.076). These results reflected that the 8^th^ edition AJCC staging system, especially the tumor stage, needs to be verified in the more clinical researches.

The 8^th^ edition AJCC staging system also has room for improvement to better predict prognosis 7, 8, 12, 19, 20. In 2008, Leije et al found that patients with cavernosal involvement had a more aggressive histology with nodal involvement and worse survival 6. Given these findings, they proposed a modified stage of spongiosal involvement or cavernosal involvement as different pT stages. Consistent with these findings, some studies have also found that spongiosal and cavernosal involvement have significant prognostic differences 18. This classification was accepted by the AJCC TNM staging system until 2018. However, the 8^th^ edition AJCC staging system also has controversy 7, 8, 12. To resolve disputes, we performed a meta-analysis to verify the prognostic value of the 8^th^ edition T stage. The results suggested that patients with CS invasion obtained a significantly better CSS than those with CC invasion but not OS.

A meta-analysis showed that CC invasion and CS invasion were significant predictors of inguinal LNM 10. This meta-analysis including one study reported that CC and CS infiltration combined were able to predict LNM. However, this meta-analysis failed to distinguish different anatomies (Figure 7 in Hu J et. al. study), and subgroup analyses were only included one or two studies (Figure 4 in Hu J et. al. study), so the results need further research. In our study, anatomical structure (CC invasion or CS invasion) and tumor stage (T2: tumor invading into CS or T3: tumor invading into CC) were calculated separately.

It is well established that clinicopathological features can predict LNM 21, 22. Some studies have shown that CS invasion or CC invasion differs from nodal involvement 23. Studies suggested that patients with CS invasion had a higher rate of LNM than those with CS invasion 6, 10, 16, 21-23. Contrary to the above points, however, spongiosal and cavernosal involvement did not show a significant correlation with nodal metastasis 8, 13, 16. The risk-adapted stratification of the European Association of Urology (EAU), which included primary tumor stage and histologic grade, was recommended to estimate the incidence rate of LNM 21. Solsona E et al. suggested that 83% of patients with pT2/3-G2/3 tumors had LNM; in contrast, pT1G1 tumors were not found to have LNM. However, occult nodal involvement will overestimate adherence to risk-adapted stratification, which would have led to 77% of patients not undergoing bilateral inguinal lymph node dissection (LND) 24.

There are some limitations to this study. 1) The number of studies was small. The analysis of OS included only two studies, which may not provide sufficient power to draw reliable conclusions. 2) The HRs were extracted from indirect comparisons. We used Engauge software to extract Kaplan-Meier curve data, which also contributed to heterogeneity. 3) Factors might impact survival, for example surgical treatments, adjuvant/or neoadjuvant therapy, nodal status, size of tumors, local vascular invasion or lymphatic invasion etc. Due to the limited number of included studies and lacked of article information, we did not perform subgroup analysis. 4) This study only involved the CSS and OS. Other outcomes will be important in future validation studies of larger and multicentre data sets. Considering these drawbacks, we firmly believe that the persuasive power of the results will greatly increase with further research.

## Conclusion

In summary, patients with CS invasion had better CSS than those with CC invasion but not OS. Our data also show that patients with CC invasion are associated with a high risk of LNM. However, due to the limited number of included studies, a larger sample size is needed for validation, which may provide a useful guide for clinicians.

## Figures and Tables

**Table 1:** Main characteristics of the studies

Group	First author	Year	Country	Recruitment period	Number	Age (median)	Follow-up (mean/median)	No. CC invasion (%)	No. CS invasion (%)	Outcomes measured	NOS score
a	Ficarra V	2005	Italy	1980-2002	175	62.0±12.2	26 (2-292)	79 (45.1)	61 (34.9)	LNM	9
	Ornellas AA	2008	Brazil	1996-2007	196	57 (25-98)	74 (1-93)	88 (44.9)	69 (35.2)	LNM	8
	Graafland NM	2010	Netherland	2001-2008	342	65 (26-96)	31 (3-91)	63 (18.4)	217 (63.4)	LNM	9
	Termini1 L	2015	Brazil	1953-2000	125	NA	NA	60 (48.0)	88 (70.4)	LNM	6
b	Amr Al-Najar	2011	Germany	1996-2008	89	31-91	1-142	18 (20.2)	23 (25.8)	LNM	8
	Kearns JT	2019	NCDB	2010-2012	912	NA	NA	524 (57.5)	378 (41.4)	LNM	6
	Akash P.	2020	India	2007- 2012	142	NA	21 (1-96)	45 (31.7)	97 (68.3)	LNM	7
c	Leijte JA	2008	Netherland	1956-2006	513	65 (21-94)	58.7 (3-303)	90 (17.5)	162 (31.6)	Survival	9
	Li ZS^1^	2018	Global	2000-2015	411	53 (24-94)	18 (1-207)	119 (29.0)	115 (28.0)	Survival	7
	Li ZS^2^	2018	Global	2000-2015	436	56 (18-93)	35.4 (1-349.7)	142 (32.6)	142 (32.6)	Survival	7
	Hölters S	2019	Germany	1992-2015	121	61.6 (25-88)	46.8 (1-176)	23 (19.0)	30 (24.8)	Survival	6
	Wang BH	2020	China	1998-2015	230	57 (47-64)	48.9 (28.1-84.7)	110 (47.8)	30 (13.0)	Survival	6

a: LNM on the basis of the anatomical structure; b: LNM on the basis of tumor stage; c: survival on the basis of tumor stage. 1: training cohort; 2: external cohort; NCDB: National Cancer Database.

**Table 2:** Pooled nodal metastasis prevalence in penile cancer by stratification studies

Group	First author	CC invasion n (%)		CS invasion n (%)	
		Nodal Metastasis (absent)	Nodal Metastasis (present)	Nodal Metastasis (absent)	Nodal Metastasis (present)
a	Ficarra V	38 (48.1)	41 (51.9)	28 (45.9)	33 (54.1)
	Ornellas AA	45 (51.4)	43 (48.7)	42 (60.9)	27 (39.1)
	Graafland NM	42 (66.7)	21 (33.3)	165 (76.0)	52 (24.0)
	Termini L	28 (50.9)	27 (49.1)	50 (60.2)	33 (39.8)
b	Amr Al-Najar	10 (55.6)	8 (44.4)	13 (56.5)	10 (43.5)
	Kearns JT	159 (54.6)	132 (45.4)	136 (66.0)	70 (34.0)
	Akash P.	7 (15.5)	38 (84.4)	24 (24.7)	73 (75.3)

a: LNM on the basis of the anatomical structure; b: LNM on the basis of tumor stage.

**Table 3:** Main characteristics of the studies

Analysis	No. of studies	HR/OR (95% CI)	Model	*P* Value	Heterogeneity	
					I2 (%)	*P* Value
**Subgroup 1 (CSS): follow-up time**						
<36 months	2	0.76 (0.54-1.06)	Fixed	0.104	13.4	0.283
≥36 months	2	0.50 (0.30-0.83)	Fixed	0.007	37.2	0.207
**Subgroup 2 (CSS): age**						
< 60	2	0.50 (0.30-0.83)	Fixed	0.007	37.2	0.207
≥ 60	2	0.76 (0.54-1.06)	Fixed	0.104	13.4	0.283
**Subgroup 3 (CSS): sample size**						
≥ 400	3	0.64 (0.43-0.95)	Random	0.028	48.8	0.142

## Abbreviations

AJCC: American Joint Committee on Cancer
CC: corpora cavernosa
CI: confidence interval
CS: corpus spongiosum
CSS: cancer-specific survival
HR: hazard ratio
LNM: lymph node metastasis
OR: odds ratio
OS: overall survival
TNM: tumor-node-metastasis
